# New Advances on Wellness Therapies Using Integrated Health Focusing on Nature

**DOI:** 10.3390/ijerph191610313

**Published:** 2022-08-19

**Authors:** Amber L. Vermeesch, Chloe Littzen-Brown, Andrew Lafrenz

**Affiliations:** 1 Department of Family and Community Nursing, School of Nursing, University of North Carolina at Greensboro, Greensboro, NC 27402, USA; 2 School of Nursing & Health Innovations, University of Portland, Portland, OR 97203, USA

## 1. Introduction

The purpose of this Special Issue is to provide you, the reader, with an overview of new advancements in wellness therapies using integrative health focusing on nature. Nature is inherently variable, and as such, we the editors do not ascribe to a single definition of nature. Experiencing the health benefits of nature is not limited to long immersions in remote nature preserves. Ongoing research highlights the benefits of even short exposure to green and blue areas. Whether looking out an office window at a single tree or on a long mindfulness retreat in a remote forest, there lies the potential to improve our mental and physical health. Depending on where you live, your proximity to water, elevation, the equator, etc., will all change the objective appearance and experience of nature. Moreover, how you perceive nature as an individual is subjective based upon your own bio-psycho-social experience with and within nature—which is why you will find variable definitions and applications of nature.

## 2. Background

What are nature-based interventions, and why are they important? In addition, why the focus on them for this Special Issue? First, nature-based interventions are not novel. Indigenous people worldwide have been practicing nature-based interventions (e.g., ethnobotany) well before what we know as contemporary science even made us aware of the impacts of nature on human health. Science is just now catching up to what indigenous people have known for years, and it is important for us to be mindful of that. Secondly, nature can be an accessible and practical intervention to promote widespread benefits across populations and individuals. Additionally, more broadly, nature-based interventions are important because they can help us, as humans, reconnect with ourselves, nature, and ultimately, each other. This is especially important now because we are at a critical crossroads in terms of nature-based disasters due to climate change, such as flooding, fires, and heat. Without our engagement with nature, ultimately, climate change will worsen, and nature as we know it will collapse. The lessons we learned from the COVID-19 pandemic should not be ignored in relation to our health and the natural world. First, improving underlying health issues should be a global priority in order for us to best navigate the uncertainty of the future of infectious disease. Nature interventions have proven to be effective strategies in improving physical health and psychosocial well-being. Now, we know the importance that optimizing one’s health has on communicable disease outcomes. Second, we must ensure that all communities have safe, healthy, and equitable access to nature no matter what their background or where they live. The articles in this issue represent the wide range of ways in which nature and our health and wellness are interconnected. The future of this field is bright, and the potential of nature to promote health and wellness is truly exciting.

## 3. Thoughts to Ponder

Before, as, and after you read the included manuscripts within this Special Issue, we ask you to ponder the following questions:

1. What is nature to you?
2. How do you benefit from nature?
3. What are three actions you can take to preserve nature and protect her?

## 4. Conclusions

If we do not practice our relationship with nature, we will lose her, and ultimately, ourselves. Despite the well-documented relationship between nature and health, there remains a lack of significant investment in not only using nature to improve health in current populations but also in protecting nature for the health of future generations. We hope that our Special Issue re-engages your interest in nature and nature-based interventions, and we welcome any comments on our Special Issue.

